# Fatty-Acid-Binding Protein 4 as a Novel Contributor to Mononuclear Cell Activation and Endothelial Cell Dysfunction in Atherosclerosis

**DOI:** 10.3390/ijms21239245

**Published:** 2020-12-03

**Authors:** Yen-Wen Wu, Ting-Ting Chang, Chia-Chi Chang, Jaw-Wen Chen

**Affiliations:** 1 Division of Cardiology, Cardiovascular Medical Center, Far Eastern Memorial Hospital, New Taipei City 220, Taiwan; wuyw0502@gmail.com; 2 School of Medicine, National Yang-Ming University, Taipei 112, Taiwan; tf0619@ym.edu.tw; 3 Department and Institute of Pharmacology, National Yang-Ming University, Taipei 112, Taiwan; 4 Healthcare and Services Center, Taipei Veterans General Hospital, Taipei 112, Taiwan; migo0427@yahoo.com.tw; 5 Cardiovascular Research Center, National Yang-Ming University, Taipei 112, Taiwan; 6 Department of Medicine, Taipei Veterans General Hospital, Taipei 112, Taiwan

**Keywords:** adhesion molecules, atherosclerosis, coronary artery disease, endothelial dysfunction, fatty acid-binding protein 4

## Abstract

Background—Elevated circulating fatty-acid-binding protein 4 (FABP4) levels may be linked with cardiovascular events. This study aimed to investigate the mechanistic role of FABP4 in atherosclerosis. Methods—We recruited 22 patients with angiographically proven coronary artery disease (CAD) and 40 control subjects. Mononuclear cells (MNCs) and human coronary endothelial cells (HCAECs) were used for in vitro study. Results—Patients with CAD were predominantly male with an enhanced prevalence of hypertension, diabetes, and smoking history. FABP4 concentrations were up-regulated in culture supernatants of MNCs from CAD patients, which were positively correlated with the patients’ age, waist–hip ratio, body mass index, serum creatinine, type 2 diabetes, and the presence of hypertension. The adhesiveness of HCAECs to monocytic cells can be activated by FABP4, which was reversed by an FABP4 antibody. FABP4 blockade attenuated the oxidized low-density lipoprotein (oxLDL)-induced expression of ICAM-1, VCAM-1, and P-selectin. FABP4 impaired the tube formation and migration via the ERK/JNK/STAT-1 signaling pathway. FABP4 suppressed phosphorylation of eNOS and expression of SDF-1 protein, both of which can be reversed by treatment with VEGF. Blockade of FABP4 also improved the oxLDL-impaired cell function. Conclusion—We discovered a novel pathogenic role of FABP4 in MNC activation and endothelial dysfunction in atherosclerosis. FABP4 may be a therapeutic target for modulating atherosclerosis.

## 1. Introduction

Fatty-acid-binding proteins (FABPs) are small cytoplasmic proteins expressed in a mainly tissue-specific manner and bind to fatty acids such as oleic and retinoic acids. FABP4, also known as adipocyte fatty-acid-binding protein, is a cytosolic fatty acid chaperone expressed in adipocytes and macrophages. Clinically, circulating FABP4 concentration, as a biomarker, can be associated with inflammation, metabolic syndrome, hypertension, and cardiovascular events [1,2,3,4]. Circulating FABP4 levels can be also associated with the clinical presence of myocardial ischemia, atherogenic dyslipidemia, and type 2 diabetes mellitus (DM) [5,6,7,8]. Serum FABP4 levels may be also related to carotid and coronary atherosclerosis, in addition to coronary artery disease (CAD) [9,10,11]. It was shown that both the expression of FABP4 and leptin can be increased in ruptured plaques with upregulated PPAR/adipocytokine signaling [12]. Furthermore, we previously showed that circulating FABP4 levels were associated with myocardial perfusion abnormalities and left ventricular function, which could be related to the development of heart failure in CAD patients [13]. Therefore, given the multiple clinical associations, the direct causal role of FABP4 in clinical atherosclerosis requires further clarification.

Previous in vivo evidence showed that obese FABP4-deficient mice would not develop insulin resistance or diabetes since they could not express tumor necrosis factor-α (TNF-α) from adipose tissues [14]. In addition, Apolipoprotein E (ApoE)-deficient mice deficient in FABP4 exerted protective effects on atherosclerosis without affecting serum lipids or insulin sensitivity [15]. It was suggested that FABP4 may contribute to metabolic disorder and atherosclerosis through its actions in both macrophages and adipocytes in mice [16,17]. However, its direct vascular effects remain unconfirmed.

Interestingly, previous in vitro studies showed that FABP4 can inhibit the insulin and endothelial nitric oxide synthase (eNOS)-signaling pathways and can lead to endothelial dysfunction in human umbilical vein endothelial cells (HUVECs) [18]. Overexpression of FABP4 increased the inflammatory cytokines and decreased phosphorylation of eNOS in human coronary artery endothelial cells (HCAECs) [19]. Both of the above-mentioned results indicate the harmful effects of FABP4. In contrast, FABP4 mRNA and protein levels can be induced by vascular endothelial growth factor (VEGF) and basic fibroblast growth factor in endothelial cells [20]. The VEGF-induced FABP4 expression can be attenuated by inhibition of the NOTCH signal transduction [21]. These observations suggest that FABP4 may be a potential target of VEGF and a positive regulator of endothelial cells proliferation. In fact, FABP4-deficient HUVECs are prone to apoptosis and have decreased migration and capillary network formation abilities. Aortic rings from FABP4-deficient mice have decreased angiogenic sprouting. FABP4 modulated intracellular signaling activation in HUVECs through P38, eNOS, and stem cell factor/c-kit signaling pathways [22]. Moreover, FABP4 can promote migration and proliferation of human coronary artery smooth muscle cells through a MAPK-dependent pathway [23]. Overall, FABP4 seems either harmful or essential to normal vascular function, effects that might vary in different vascular cells under different conditions. Its mechanistic role in disease conditions such as atherosclerosis remain poorly understood. Given the controversial evidence of FABP4 in vascular cell function, this study aimed to clarify the potential mechanistic effects of FABP4 in clinical atherosclerosis.

## 2. Results

### 2.1. Up-Regulated FABP4 Secretion and Enhanced Endothelial Adhesiveness of MNCs from CAD Patients

**Figure 1:**
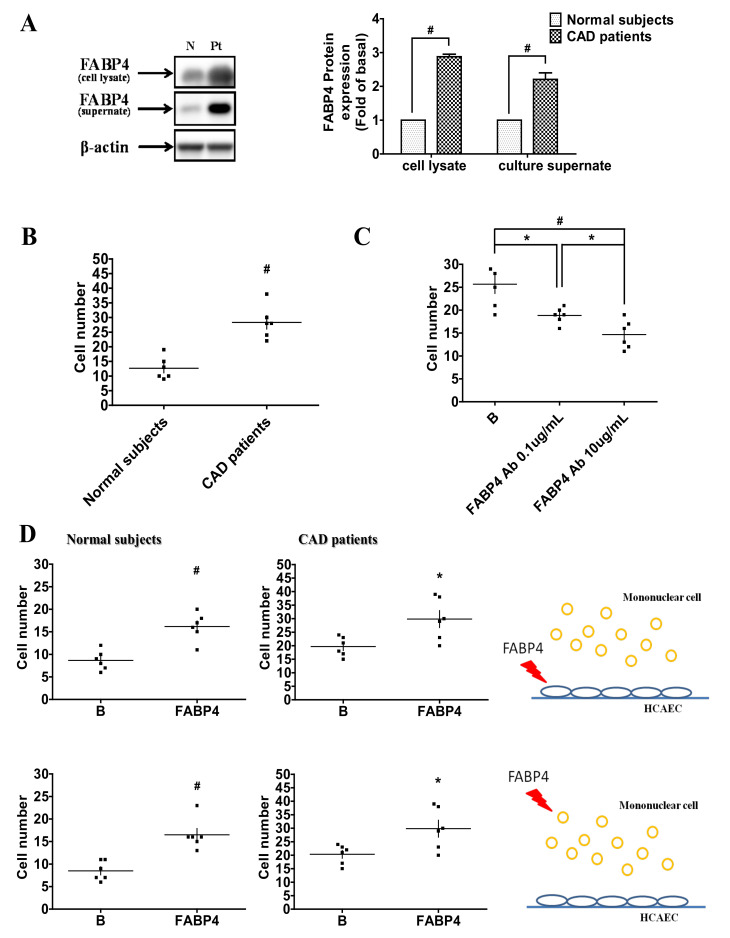
Coronary artery disease (CAD) patients had higher FABP4 levels and adhesiveness of human coronary artery endothelial cells (HCAECs) to mononuclear cells (MNCs). MNCs were cultured for 4 days. The FABP4 expression in the supernatants and cell lysates (**A**). The adhesiveness of MNCs from CAD patients and normal subjects (**B**). The adhesiveness of MNCs from CAD patients can be inhibited by FABP4 neutralizing antibody (**C**). Exogeneity of FABP4 on HCAECs or MNCs from CAD patients and normal subjects increased their adhesiveness abilities (**D**). N represents normal subjects; Pt represents CAD patients; B represents baseline. *N* = 6 in each experiment. Data are presented as means ± standard errors. Statistical analyses were performed using Student’s *t* test. Data were considered statistically significant when the *p*-value was <0.05. * *p* < 0.05, # *p* < 0.01.

There was no significant difference in plasma FABP4 and oxidized low-density lipoprotein (LDL) concentrations between CAD patients and control subjects. The FABP4 levels in the culture medium and cell lysates were significantly higher in CAD patients than those in control subjects (Table 1, Figure 1A). Furthermore, FABP4 levels in mononuclear cell (MNC) culture supernatants were positively correlated with age, waist–hip ratio (WHR), history of hypertension, DM, and serum creatinine levels, but negatively correlated with physical activity and high-density lipoprotein (HDL)-C. After multivariable adjustment, only hypertension was independently associated with FABP4 concentrations (Table 2).

Additionally, increased adhesiveness of MNCs from CAD patients to HCAECs was observed (Figure 1B). The enhanced endothelial adhesiveness of MNCs can be inhibited by the treatments of FABP4 neutralizing antibody in a dose-dependent manner (Figure 1C). Exogeneity of FABP4 on the MNCs from CAD patients and control subjects or HCAECs directly increased their adhesiveness (Figure 1D).

### 2.2. FABP4 Inhibition Decreased the Oxidized-LDL-Induced Adhesion of MNCs Via Down-Regulating the Integrin β2, Integrin α4, and PSGL-1 Expression

**Figure 2:**
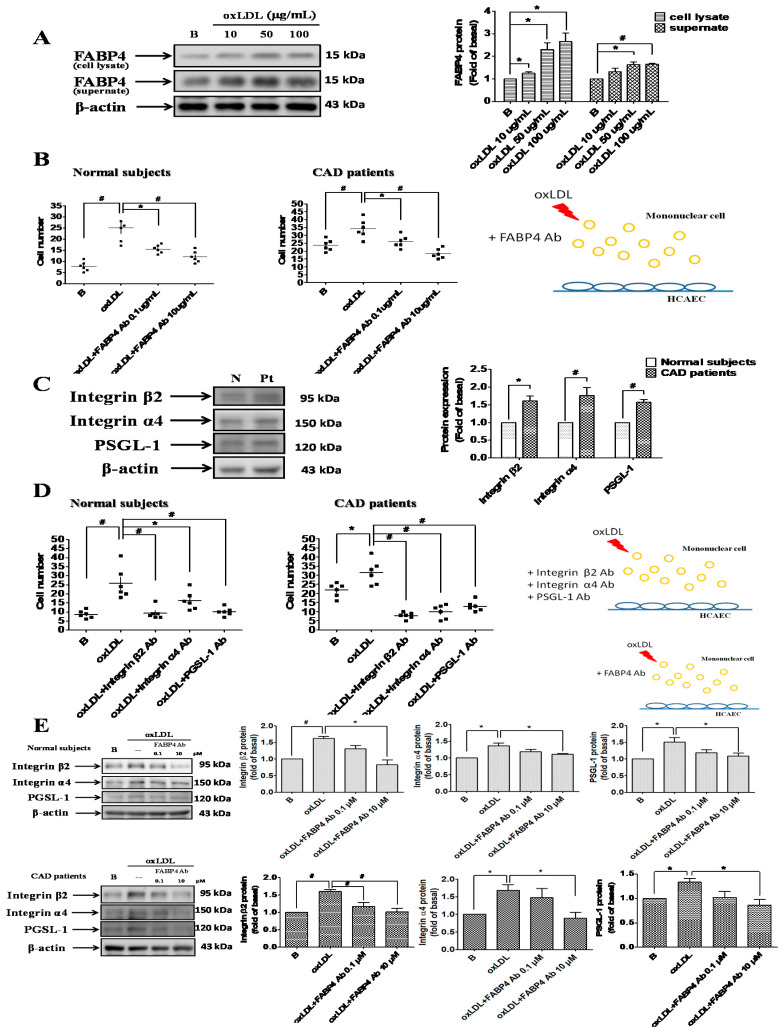
FABP4 inhibition suppressed the oxidized-LDL-induced adhesiveness of MNCs by modulating the adhesion molecules. Oxidized-LDL enhanced the FABP4 expression in MNCs from control subjects (**A**). The oxidized-LDL (50 µg/mL)-induced adhesiveness of MNCs was reversed by FABP4 neutralizing antibody (**B**). The expression of adhesion molecules, such as intergrin β2, integrin α4, and P-selectin glycoprotein ligand 1 (PSGL-1), was higher in MNCs from CAD patients (**C**). The oxidized-LDL-induced adhesiveness of MNCs was reversed by integrin β2, integrin α4, and PSGL-1 neutralizing antibodies (**D**). FABP4 neutralizing antibody reduced the oxidized-LDL-induced integrin β2, integrin α4, and PSGL-1 expression in MNCs (**E**). N represents normal subjects; Pt represents CAD patients; B represents baseline. N = 6 in each experiment. Data are presented as means ± standard errors. Statistical analyses were performed using Student’s *t* test. Data were considered statistically significant when the *p*-value was <0.05. * *p* < 0.05, # *p* < 0.01.

Oxidized-LDL increased FABP4 expression in MNCs from control subjects (Figure 2A). The adhesiveness levels of MNCs from CAD patients and control subjects were further enhanced by oxidized-LDL, and these enhancements were attenuated by FABP4 neutralizing antibody (Figure 2B). The expression of adhesion molecules, such as integrin β2, integrin α4, and PSGL-1 were higher in MNCs from CAD patients (Figure 2C). The oxidized-LDL-induced adhesiveness of MNCs was blocked by the neutralizing antibodies of integrin β2, α4, and PSGL-1 (Figure 2D). Furthermore, FABP4 neutralizing antibody reduced the oxidized-LDL-induced integrin β2, α4, and PSGL-1 expression in MNCs from CAD patients or control subjects (Figure 2E). Overall, these results indicated that FABP4 inhibition can suppress the oxidized-LDL-induced adhesiveness of MNCs through down-regulating the integrin β2, α4, and PSGL-1 expression.

### 2.3. FABP4 Inhibition Decreased the Oxidized-LDL-Induced Adhesion of HCAECs Via Down-Regulating the ICAM-1, VCAM-1, and P-Selectin Expression

**Figure 3:**
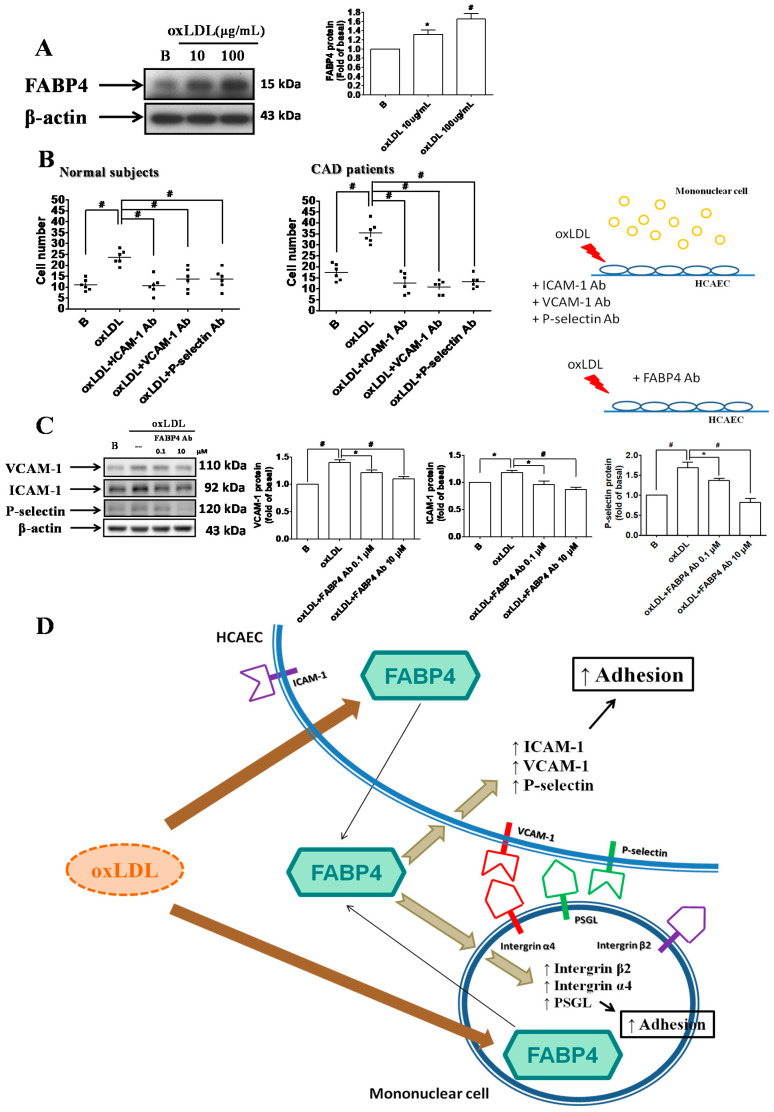
FABP4 inhibition suppressed the oxidized-LDL-induced adhesiveness of HCAECs by modulating the adhesion molecules. Oxidized-LDL enhanced the FABP4 expression in HCAECs (**A**). The oxidized-LDL-induced adhesiveness of HCAECs was reversed by ICAM-1, VCAM-1, and P-selectin neutralizing antibodies (**B**). FABP4 neutralizing antibody reduced the oxidized-LDL-induced ICAM-1, VCAM-1, and P-selectin expression in HCAECs (**C**). The proposed mechanisms are illustrated (**D**). *N* = 6 in each experiment. Data are presented as means ± standard errors. Statistical analyses were performed using Student’s *t* test. Data were considered statistically significant when the *p*-value was <0.05. * *p* < 0.05, # *p* <0.01.

Oxidized-LDL enhanced the FABP4 expression in HCAECs (Figure 3A). Oxidized-LDL increased the adhesiveness of HCAECs to MNCs from CAD patients and from control subjects. In addition, the oxidized-LDL-induced adhesiveness of HCAECs was reversed by ICAM-1, VCAM-1, and P-selectin neutralizing antibodies (Figure 3B). Furthermore, FABP4 inhibition can reduce the oxidized-LDL-induced expression of ICAM-1, VCAM-1, or P-selectin in HCAECs (Figure 3C). In summary, FABP4 inhibition can suppress the oxidized-LDL-induced adhesiveness of HCAECs through down-regulating the ICAM-1, VCAM-1, or P-selectin expression. The proposed mechanisms of FABP4 in atherogenesis are illustrated in Figure 3D.

### 2.4. FABP4 Impaired the Functions of HCAECs via the ERK/JNK/STAT-1 Signaling Pathways

**Figure 4:**
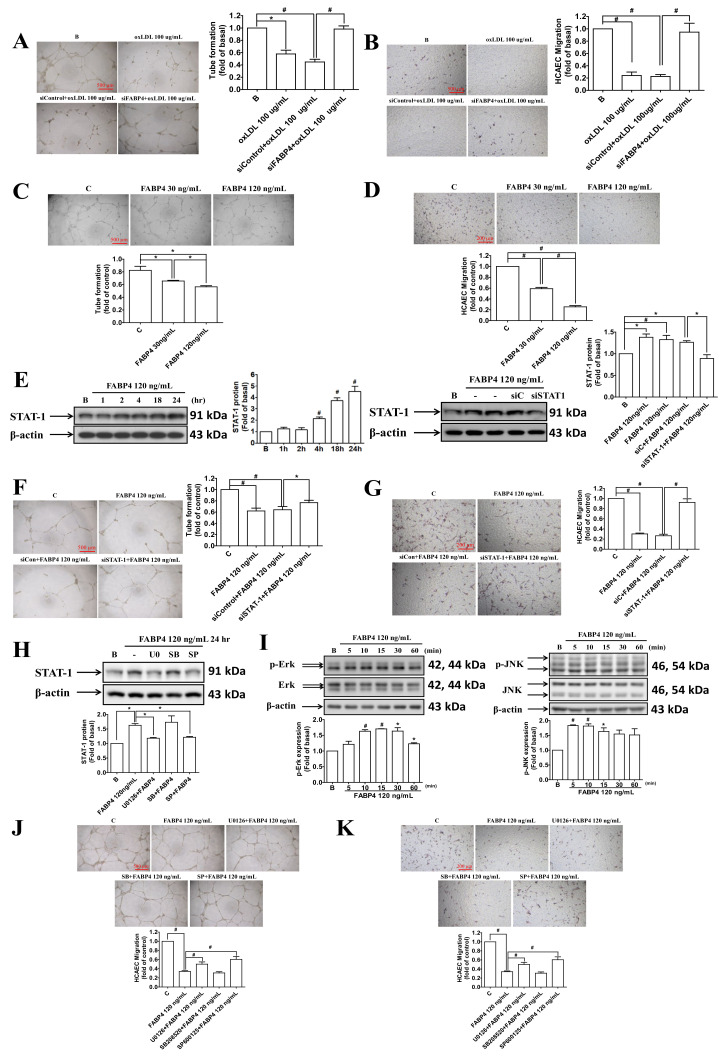
The effects and signaling pathways of FABP4 in HCAECs. The administration of FABP4 siRNA recovered the oxidized-LDL-impaired tube formation and migration abilities in HCAECs (**A**,**B**). Treatment with exogenous FABP4 directly impaired the tube formation and migration abilities of HCAECs (**C**,**D**). Treatment of FABP4 induced STAT-1 expression in HCAECs, which was reduced by STAT-1 siRNA (siSTAT-1) but not siControl (siC) (**E**). The FABP4-impaired tube formation and migration abilities of HCAECs can be recovered by siSTAT-1 (**F**,**G**). FABP4-induced STAT-1 expression can be reduced by U0126 (10 µM) and SP600125 (3 µM) but not SB208520 (5 µM) (**H**). Exogenous FABP4 can induce the phosphorylation of ERK (p-ERK) and JNK (p-JNK) (**I**). The FABP4-impaired tube formation and migration abilities of HCAECs can be recovered by U0126 and SP600125 but not by SB208520 (**J**,**K**). U0126 (an ERK inhibitor), SB208520 (a p38 inhibitor), and SP600125 (a JNK inhibitor). N = 3 in each experiment. Data are presented as means ± standard errors. Statistical analyses were performed using Student’s *t* test. Data were considered statistically significant when the *p*-value was <0.05. * *p* < 0.05, # *p* <0.01.

We used FABP4 siRNA to investigate the endogenous role of FABP4 in HCAECs. After the blockade of endogenous FABP4, the oxidized-LDL-impaired tube formation and migration abilities were recovered (Figure 4A,B). Administration of the exogenous FABP4 directly impaired the tube formation and migration abilities of HCAECs (Figure 4C,D). The STAT-1 protein expression was induced by exogeneity of FABP4, which was attenuated by the siRNA of STAT-1 (Figure 4E). The impaired tube formation and migration abilities of HCAECs by FABP4 was recovered by siSTAT-1 (Figure 4F,G). To further confirm the signaling pathways mediated by FABP4, U0126 (an ERK inhibitor), SB208520 (a p38 mitogen-activated protein kinase inhibitor), and SP600125 (a JNK inhibitor) were used in the following experiments. The FABP4-induced STAT-1 expression was reduced by U0126 and SP600125 but not by SB20852 (Figure 4H). In addition, exogeneity of FABP4 can activate the phosphorylation of ERK and JNK (Figure 4I). In the functional assay, the impaired tube formation and migration abilities of HCAECs by FABP4 were recovered by U0126 and SP600125 but not by SB208520 (Figure 4J,K). The above data indicated that FABP4 impaired the endothelial function through the ERK/ JNK/ STAT-1 signaling pathways.

### 2.5. VEGF Reversed the FABP4-Imapired Functions of HCAECs via Up-Regulating the eNOS Phosphorylation and SDF-1 Expression

**Figure 5:**
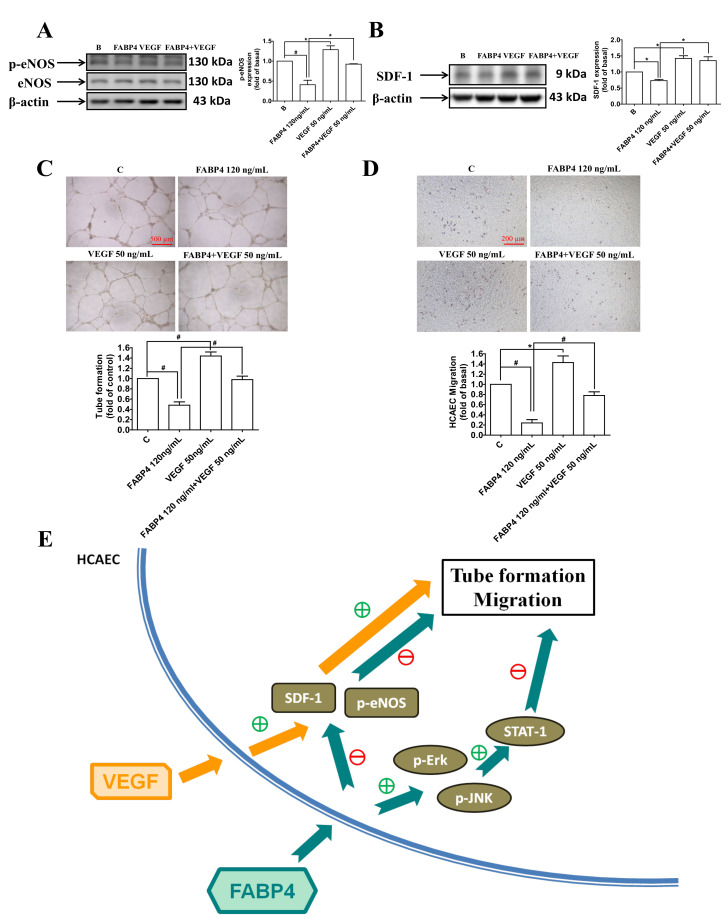
Vascular endothelial growth factor (VEGF) reversed the FABP4-imapired functions of HCAECs by modulating the pro-angiogenic proteins. FABP4 decreased the phosphorylation of endothelial nitric oxide synthase (p-eNOS) and stromal cell-derived factor 1 (SDF-1) in HCAECs. Both p-eNOS and SDF-1 levels were recovered in the VEGF-treated group (**A**,**B**). FABP4 impaired tube formation and migration abilities of HCAECs, and these functions were reversed by the administration of VEGF (**C**,**D**). The proposed mechanisms are illustrated (**E**). N = 3 in each experiment. Data are presented as means ± standard errors. Statistical analyses were performed using Student’s *t* test. Data were considered statistically significant when the *p*-value was <0.05. * *p* < 0.05, # *p* < 0.01

The levels of phospho-eNOS and SDF-1 were reduced by FABP4, and were recovered by the administration of VEGF (Figure 5A). The FABP4-impaired tube formation and migration abilities of HCAECs were also reversed by VEGF treatments (Figure 5C,D). These observations suggested that FABP4 impaired the endothelial functions through the eNOS and SDF-1 signaling pathways, which were rescued by VEGF.

Overall, FABP4 can lead to endothelial dysfunction through different mechanisms, including ERK/ JNK/ STAT-1, eNOS, and SDF-1 signaling pathways. The potential mechanisms of FABP4 in modulating endothelial dysfunction are summarized in Figure 5E.

## 3. Discussion

Given that the interaction between MNCs and HCAECs may be essential for atherosclerosis, in this study, there are some new findings about the direct role of FABP4 in atherosclerosis. First, FABP4 plays a pivotal role in modulating the adhesiveness of HCAECs to MNCs. Second, FABP4 could be induced by oxidized-LDL in both MNCs and HCAECs. Third, the direct inhibition of FABP4 could reverse the oxidized-LDL-induced adhesion between MNCs and HCAECs through the downregulation of the integrin β2, α4, and PSGL-1 pathways in MNCs and the ICAM-1, VCAM-1, and P-selectin pathways in HCAECs. Finally, FABP4 directly impaired the functions of HCAECs through the up-regulation of the ERK/JNK/STAT-1 signaling and the down-regulation of the eNOS and SDF-1 pathways. In summary, although previous data are mainly related to HUVECs or macrophages, this study provides novel mechanisms of FABP4 in coronary endothelial cell injury and the atherosclerotic interaction between different vascular cells. Our findings support the key role of FABP4 as a potential therapeutic target for atherosclerosis.

CAD patients were predominantly male with higher prevalence of hypertension and DM. In addition, CAD patients had higher hip circumference and WHR but lower total cholesterol, LDL-C, and HDL-C than did controls. Elevated FABP4 levels were observed in MNCs from CAD patients compared to those from control subjects although there were no significant differences in plasma FABP4 and oxidized-LDL concentrations between the two groups. In comparison to the control group, the CAD group took more medications, and previous research showed that treatment with atorvastatin can reduce serum FABP4 levels in patients with hyperlipidemia [24,25]. Accordingly, the decreased levels of cholesterol profiles and FABP4 concentrations in the CAD group might be due to statin usage. Furthermore, the enhanced FABP4 concentrations were positively correlated with age, WHR, history of hypertension, DM, and serum creatinine levels, but negatively correlated with physical activity and HDL-C. However, only hypertension can be an independent predictor for FABP4 levels.

FABP4 is a gene that is highly up-regulated in macrophages in response to oxidized-LDL by the activation of peroxisome proliferator-activated receptor gamma (PPARgamma) transcription factors, since the endogenous PPARgamma ligands can induce FABP4 expression in macrophage/foam cells [26]. In addition, overexpression of the FABP4 gene in macrophages enhances the accumulations of cholesterol and triglyceride [27]. Lack of FABP4 protects ApoE-deficient mice against atherosclerosis [15]. In our study, we showed that FABP4 can increase the adhesiveness of HCAECs to MNCs, which can be an early sign of atherogenesis. Furthermore, FABP4 protein expression can be induced by oxidized-LDL. The direct FABP4 blockades can attenuate the oxidized-LDL-induced adhesion between MNCs and HCAECs through down-regulating different adhesion molecules in MNCs and HCAECs. Actually, increased circulating FABP4 levels were associated with endothelial dysfunction in patients with type 2 diabetes [28]. Administration of a FABP4 inhibitor improved endothelial function in ApoE-deficient mice and in cultured human microvascular endothelial cells with enhanced phosphorylated eNOS and NO production [29]. These results were consistent with those of previous research; we further discovered that enhanced FABP4 levels were associated with CAD patients and can be a predictor for hypertension. Most importantly, we determined that FABP4 can lead to endothelial dysfunction by ERK/JNK/STAT-1 signaling, eNOS, and SDF-1 pathways.

The increased peroxisome proliferator-activated receptor gamma activity in FABP4-deficient macrophages was accompanied by a stimulated liver X receptor α-ATP-binding cassette transporter A1-mediated cholesterol efflux pathway. In addition, FABP4-deficient macrophages showed reduced IkappaB kinase and NF-kappaB activity, leading to suppressed inflammation such as reduced cyclooxygenase-2 and inducible nitric-oxide synthase expression, in addition to inflammatory cytokines [30]. In contrast, FABP4 can modulate lipopolysaccharide-induced inflammatory responses in macrophages by a positive feedback loop involving c-Jun NH2-terminal kinases and activator protein-1 [31]. As a result, the effect and mechanism of FABP4 in MNCs and HCAECs on the inflammation-related pathway in our in vitro models still needs further investigation.

Toll-like receptors can initiate inflammatory responses in atherosclerosis and STAT-1 is involved in the signaling pathways mediated by toll-like receptor 4, resulting in increased expression of several pro-inflammatory and pro-atherogenic mediators. Accordingly, STAT1 inhibitor may be a potential therapeutic strategy for atherosclerosis [32]. Moreover, SDF-1 and CXCR4 may affect native atherogenesis by modulating atherosclerosis-relevant cellular functions [33]. On the other hand, although angiogenesis is related to the thickness and stability of the plaque, the formation of atherosclerosis may be affected independently of the angiogenesis in the plaque. Inflammation and endothelial adhesion molecules may be more important [34]. In this study, FABP4 could enhance endothelial adhesion and induce endothelial dysfunction by activating ERK/JNK/STAT-1 signaling pathways and inhibiting both eNOS and SDF-1 expression in HCAECs. In addition, the impaired functions of HCAECs can be reversed by VEGF treatments.

In conclusion, we showed that FABP4 can not only cause atherogenesis through the activation of the adhesion molecules, including integrin β2, α4, and PSGL-1 in MNCs and ICAM-1, VCAM-1, and P-selectin in HCAECs, but can also lead to endothelial dysfunction through the activation of the STAT-1 signaling pathways, which in turn may impair the expression/phosphorylation of pro-angiogenic molecules, such as eNOS and SDF-1. Although the sample size of patients with coronary artery disease was rather modest and additional studies with larger numbers of subjects or in vivo experiments are required to confirm the findings, our results suggest the novel and comprehensive roles of FABP4 in the pathogenesis of atherosclerosis from basic to clinical. While additional RT PCR analysis of respected genes could be helpful to support protein analysis in this study, further studies may be also warranted to verify if the blockade of FABP4 might be a promising therapeutic strategy for clinical atherosclerosis.

## 4. Materials and Methods

### 4.1. Patients and Study Design

The case–control study aimed to enroll patients with angiographic-documented significant CAD (angiographic stenosis ≥50% in the main branch of major coronary arteries) and apparently healthy or patent coronary controls. The study was approved by the research ethics review committee of Far Eastern Memorial Hospital (FEMH-IRB-104092-E, 11082015), and subjects gave written informed consent before entering the study. Demographic and clinical data were obtained at enrollment. The human study was approved by the institute research committee and conformed with the Declaration of Helsinki. A total of 22 patients with CAD and 40 control subjects were included.

### 4.2. Characteristics of Study Population

A total of 22 patients with CAD and 40 control subjects were included. Between the two groups, patients with CAD were predominantly male (86%) with a mean ± SD age of 66 ± 10 with significantly higher prevalence of family history of CAD and DM, in addition to the presence of hypertension and type 2 DM, and were more likely current smokers, compared to controls. Compared to the control subjects, CAD patients had increased hip circumference, waist–hip ratio (WHR), and systolic blood pressure, but reduced serum total cholesterol, low-density lipoprotein-cholesterol (LDL-C), and high-density lipoprotein-cholesterol (HDL-C) levels. Furthermore, the CAD patients had significantly more medicine usages, such as beta-blockers, angiotensin converting enzyme inhibitors/angiotensin II receptor blockers (ACEIs/ARBs), calcium channel blockers (CCBs), and statins (Table 1).

### 4.3. Blood Sample Collections

In all subjects, blood samples were collected in the morning under a fasting state. Human MNCs were isolated from peripheral blood and cultured for 4 days. In detail, the circulating human mononuclear cells (MNCs) were isolated from the peripheral blood by density gradient centrifugation with Histopaque-1077 (1.077 g/mL, Sigma, St. Louis, MO, USA). The MNCs were cultured in RPMI1640 medium (Corning, Manassas, VA, USA) with 10% FBS for 4 days, and the media were then collected. Routine serum biochemical testing using standard detection methods was undertaken in a central lab.

### 4.4. Cell Culture

Primary human coronary artery endothelial cells (HCAECs) (ScienCell, Carlsbad, CA, USA) were cultured in fibronectin-coated plates with endothelial cell medium (ECM; ScienCell, Carlsbad, CA, USA) containing 5% fetal bovine serum, 1% endothelial cell growth supplement, and 1% penicillin/streptomycin solution at 37 °C in a humidified atmosphere of 5% CO_2_/95% air.

### 4.5. Measurement of FABP4 Concentrations in Plasma and in Culture Supernatants of Mononuclear Cells from Subjects

In both CAD patients and healthy subjects (as the control), plasma and the culture supernatants of circulating MNCs were collected. The concentrations of FABP4 were determined by ELISA (R&D system, Minneapolis MN, USA) according to manufacturer’s instructions.

### 4.6. Western Blotting

The whole cell lysates were subjected to SDS-polyacrylamide (10–12%) gel electrophoresis and transferred to a PVDF membrane. Membranes were probed with monoclonal antibody directed to β-actin, vascular cell adhesion molecule-1 (VCAM-1), intercellular adhesion molecule-1 (ICAM-1), signal transducer and activator of transcription 1 (STAT-1), phosphorylation of endothelial nitric oxide synthase (p-eNOS), stromal cell-derived factor 1 (SDF-1), FABP4 (Cell Signaling, Beverly, MA, USA), P-selectin, intergrin β2, integrin α4, and P-selectin glycoprotein ligand 1 (PSGL-1) (Santa Cruz, Dallas, TX, USA). The immunoreactive bands detected by enhanced chemiluminescence reagents were developed by Hyperfilm-ECL (PerkinElmer, Waltham, MA, USA).

### 4.7. Cellular Viability Assay

The HCAECs and MNCs were incubated with FABP4 (30, 60, 120, 240 ng/mL; Cayman, Ann Arbor, MI, USA) for 24 h. Then, 5 mg/mL of MTT (Sigma, St. Louis, MO, USA) was added the cells were incubated at 37 °C for 4 h. After the supernatant was removed, 100 μL of DMSO (Sigma, St. Louis, MO, USA) was added to each well. The absorbance at 570 nm was determined.

### 4.8. Tube Formation Assay

Tube formation assay was performed to assess the vasculogenesis ability of HCAECs. In vitro tube formation assay was performed with the In Vitro Angiogenesis Assay Kit (Millipore, Temecula, CA, USA). The protocol was undertaken according to the manufacturer’s instructions. In brief, ECMatrix gel solution was thawed at 4 °C overnight, then mixed with ECMatrix diluent buffer and placed in a 96-well plate at 37 °C for 1 h to allow the matrix solution to solidify. HCAECs were harvested as described above with trypsin/EDTA and then 1 × 10^4^ HCAECs were placed in the matrix solution with ECM medium and incubated at 37 °C for 16 h. Tubule formation was inspected under an inverted light microscope (×40). Four representative fields were taken and the average of the total area of complete tubes formed by cells was compared by Image-Pro Plus software (MEDIA CYBERNETICS, Rockville, MD, USA).

### 4.9. Migration Assay

The migration of HCAECs was evaluated by Transwell (Corning, Tewksbury, MA, USA) assay. In brief, isolated HCAECs were detached as described above with trypsin/EDTA and then 4 × 10^4^ cells were placed in the upper chamber of 24-well Transwell plates with a polycarbonate membrane (8 μm pores) with serum-free medium. Culture medium containing VEGF (50 ng/mL) was placed in the lower chamber. After incubation for 24 h, the membrane was washed with PBS and fixed with 4% paraformaldeyde. The membrane was then stained using hematoxylin solution. The upper side of the cells was removed. The magnitude of HCAEC migration was evaluated by counting the migrated cells in 6 random high-power (×100) microscope fields.

### 4.10. The Transfection of FABP4 or STAT-1 siRNA

Transfection of FABP4 or STAT-1 siRNA duplexes (Dharmacon, Lafayette, CO, USA) was performed using Lipofectamine RNAiMax (Invitrogen, Carlsbad, CA, USA) according to the manufacturer’s protocols.

### 4.11. Adhesion Assay

The MNCs from study subjects were labeled with 10 μM BCECF-AM at 37 °C for 1 h in RPMI-1640 medium (Corning, Manassas, VA, USA). Confluent HCAECs were incubated with MNCs (5 × 10^5^ cells/mL) at 37 °C for 1 h. Nonadherent MNCs were removed and the adherent MNCs were counted under four fields per 200× high-power field using a fluorescent microscope (Zeiss, Axiovert 200 M, Oberkochen, Germany). Six randomly chosen high-power fields were counted per well.

### 4.12. Statistical Analysis

Data are presented as means ± standard errors for continuous variables or percentages for categorical variables. Intergroup comparisons were performed by Student’s *t* test or ANOVA, and proportions were compared using the chi-square test. The relationships of parameters with the presence of CAD were determined with logistic regression and multivariate models. Data were considered statistically significant when the *p*-value was <0.05.

## Figures and Tables

**Table 1:** The clinical characteristics of the study population.

	CAD	Controls	*p*
	*n* = 22	*n* = 40	
Age (year)	66 ± 10	34 ± 7	<0.0001
Male gender (n, %)	86%	40%	0.0022
Waist (cm)	85 ± 10	80 ± 13	0.15
Hip (cm)	85 ± 9	91 ± 10	0.0367
WHR	1.00 ± 0.09	0.89 ± 0.11	0.0002
BH (cm)	164 ± 10	167 ± 9	0.22
BW (kg)	69 ± 12	68 ± 14	0.81
BMI	25.5 ± 3.7	24.1 ± 3.6	0.16
Systolic BP (mmHg)	140 ± 17	126 ± 15	0.0013
Diastolic BP (mmHg)	78 ± 12	75 ± 11	0.32
HR (bpm)	76 ± 13	72 ± 8	0.09
Family History			
MI/CVA/SD (%)	23%	18%	0.63
DM (%)	18%	15%	0.75
Clinical History			
Hypertension (%)	82%	23%	<0.0001
Type 2 DM (%)	41%	0%	<0.0001
CVA (ischemic/TIA) (%)	5%	0%	0.18
PAOD (%)	5%	0%	0.18
Smoking (%)	41%	13%	0.0099
Alcohol consumption (%)	27%	10%	0.08
Physical Activity (≥30 min) (%)	9%	48%	0.0018
Medication			<0.0001
Beta-blockers (%)	64%	5%	
ACEi/ARB (%)	36%	3%	
CCB (%)	27%	3%	
Statin (%)	64%	3%	
Lab data			
T-CHO (mg/dl)	144 ± 28	179 ± 29	<0.0001
HDL-C (mg/dl)	47 ± 18	64 ± 18	0.0036
LDL-C (mg/dl)	85 ± 22	105 ± 31	0.002
Triglyceride (mg/dl)	118 ± 65	100 ± 55	0.27
Fasting glucose (mg/dl)	114 ± 41	92 ± 16	0.0069
ALT (IU/L)	29 ± 24	22 ± 14	0.20
Cre (mg/dl)	1.6 ± 2.1	0.8 ± 0.2	0.0256
UA (mg/dl)	5.6 ± 1.1	6.2 ± 1.6	0.14
Mean Adhesion Cell No. (n)	22.6 ± 21.2	38 ± 29	0.0306
FABP4 concentration			
Plasma (pg/mL)	5240 ± 1019	5052 ± 1037	0.5
Supernatant (pg/mL)	3365 ± 1500	2061 ± 829	0.0001
oxLDL (U/L)	50.2 ± 16.8	52.7 ± 14.3	0.54

ALT: Alanine transaminase; ACEi/ARB: Angiotensin converting enzyme inhibitor/angiotensin II receptor blocker; BH: body height; BMI: body mass index; BP blood pressure; BW: body weight; CAD: coronary artery disease; CCB: calcium channel blocker; CVA: cerebrovascular accident; Cre: creatinine; DM: diabetes mellitus; FABP4: fatty acid-binding protein 4; HDL-C: high density lipoprotein-cholesterol; HTN: hypertension; HR: heart rate; LDL-C: low density lipoprotein-cholesterol; LVEF: left ventricular ejection fraction; MI: myocardial infarction; PAOD: peripheral arterial occlusive disease; oxLDL: oxidized low density lipoprotein; SD: sudden death; UA: uric acid; WHR: waist–hip ratio; T-CHO: total cholesterol; TIA: transient ischemic accident. Data are presented as means ± standard errors for continuous variables or percentages for categorical variables. Intergroup comparisons were performed by Student’s *t* test or ANOVA, and proportions were compared using the chi-square test. The relationships of parameters with the presence of CAD were determined with logistic regression and multivariate models. Data were considered statistically significant when the *p*-value was <0.05.

**Table 2:** Relationships between supernatant FABP4 concentration and clinical parameters.

	Univariate		Multivariate	
	*r*	*p*	ß	*p*
Age	0.5167	<0.0001		
WHR	0.5853	0.0002		
HTN	0.4642	<0.0001	727.6	0.008
DM	0.3942	0.0002		
Physical Activity	−0.514	<0.0001		
HDL-C	−0.3321	0.036		
Cre	0.4839	<0.0001		

Cre: creatinine; DM: diabetes mellitus; FABP4: fatty acid-binding protein 4; HDL-C: high density lipoprotein-cholesterol; HTN: hypertension; WHR: waist–hip ratio. Data are presented as means ± standard errors for continuous variables or percentages for categorical variables. Intergroup comparisons were performed by Student’s *t* test or ANOVA, and proportions were compared using the chi-square test. The relationships of parameters with the presence of CAD were determined with logistic regression and multivariate models. Data were considered statistically significant when the *p*-value was <0.05.
